# bHLH-regulated routes in anther development in rice and Arabidopsis

**DOI:** 10.1590/1678-4685-GMB-2023-0171

**Published:** 2024-02-19

**Authors:** Francieli Ortolan, Thomaz Stumpf Trenz, Camila Luiza Delaix, Fernanda Lazzarotto, Marcia Margis-Pinheiro

**Affiliations:** 1 Universidade Federal do Rio Grande do Sul, Programa de Pós-Graduação em Genética e Biologia Molecular, Departamento de Genética, Porto Alegre, RS, Brazil.; 2 Universidade Federal do Rio Grande do Sul, Centro de Biotecnologia, Programa de Pós-Graduação em Biologia Celular e Molecular, Porto Alegre, RS, Brazil.

**Keywords:** Anther, male sterility, bHLH, transcription factor

## Abstract

Anther development is a complex process essential for plant reproduction and crop yields. In recent years, significant progress has been made in the identification and characterization of the bHLH transcription factor family involved in anther regulation in rice and Arabidopsis, two extensively studied model plants. Research on bHLH transcription factors has unveiled their crucial function in controlling tapetum development, pollen wall formation, and other anther-specific processes. By exploring deeper into regulatory mechanisms governing anther development and bHLH transcription factors, we can gain important insights into plant reproduction, thereby accelerating crop yield improvement and the development of new plant breeding strategies. This review provides an overview of the current knowledge on anther development in rice and Arabidopsis, emphasizing the critical roles played by bHLH transcription factors in this process. Recent advances in gene expression analysis and functional studies are highlighted, as they have significantly enhanced our understanding of the regulatory networks involved in anther development.

## Introduction

Anther development is a complex process of tight regulation of gene expression towards the differentiation and maturation of cells, including the pollen mother cells that give rise to the pollen grains. The anther development is a critical step in plant reproduction, which is fundamental for the survival and maintenance of plant species (Zhang and Wilson, 2009). This developmental process significantly impacts crop yields in many plant species. Hence, understanding the regulation of anther development can help plant breeders develop crops with higher yields. Also, anther development involves the differentiation of several cell types, such as tapetal, microsporocyte, and pollen cells, providing valuable insights into the mechanisms that regulate cell differentiation in plants (Ma, 2005; Wilson and Zhang, 2009). Overall, comprehending the regulation of anther development is crucial for advancing our understanding of plant biology and developing novel approaches to enhance crop yields and breeding. 

Anther cells display precise specification and functionality, carefully orchestrated by a cascade of transcription factors. Notably, many of these regulatory proteins are classified as members of the basic helix-loop-helix (bHLH) family, highlighting the importance of this gene family in the development of microsporangia. These transcription factors control the regulation of gene expression involved in the differentiation and maturation of cells in a precise spatiotemporal manner (Heim et al., 2003; Zuo et al., 2023). The availability of male-sterile mutants in Arabidopsis has allowed significant progress in identifying key candidates acting on anther developmental regulatory network in this model plant (Sanders et al., 1999; Ma, 2005). Overall, the loss of function of such bHLH-encoding genes negatively impacts plant reproduction due to poor morphology and the production of non-functional pollen. On the other hand, the induction of male sterility in crop plants compels the identification of orthologous genes involved in anther development in plants, such as rice and maize, among other crops (Carretero-Paulet et al., 2010).

This review focuses on the bHLH transcription factors involved in the main stages of anther development in rice and Arabidopsis, as well as their interactors and target genes. We discuss recent advances made in this field, providing a comprehensive overview of the topic.

## Anther development stages

**Figure 1 -:**
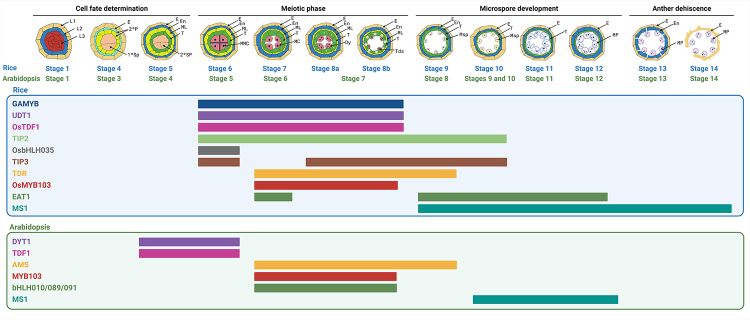
The expression of TFs in the anther development in rice and Arabidopsis. Schematic representation of anther development classified into 14 stages in rice and Arabidopsis, based on Sanders et al. (1999), Itoh et al. (2005), and Zhang et al. (2011). Phases in this representation highlight the primary events occurring in respective stages. The stages related to cell fate determination range from 1 to 5 in rice and 1 to 4 in Arabidopsis. The meiotic phase includes stages 6, 7, 8a, and 8b in rice, and stages 5, 6 and 7 in Arabidopsis. The microspore maturation phase spans stages 9 to 12 in rice and 8 to 12 in Arabidopsis. Finally, anther dehiscence takes place during stages 13 and 14 in both rice and Arabidopsis. The color of each gene is the same between orthologs in rice and Arabidopsis. L1, L2, L3: cell layers in stamen primordia; E: epidermis; 2°P: secondary parietal layers; 1°Sp: primary sporogenous cells; 2°Sp: secondary sporogenous cells; En: endothecium; ML: middle layer; T: tapetum; MMC: microspore mother cell; MC: meiotic cell; Dy: dyad cell. Tds: tetrads. Msp: microspore parietal cell. MP: mature pollen. GAMYB: GIBBERELLIN MYB GENE; UDT1: UNDEVELOPED TAPETUM1; TDF1: DEFECTIVE IN TAPETAL DEVELOPMENT AND FUNCTION1; TIP2: bHLH142/TDR INTERACTING PROTEIN2; TIP3: TDR INTERACTING PROTEIN3; TDR: TAPETUM DEGENERATION RETARDATION; EAT1: ETERNAL TAPETUM1; MS1: MALE STERILITY1; DYT1: DYSFUNCTIONAL TAPETUM1; AMS: ABORTED MICROSPORES.

The processes of anther development in rice and Arabidopsis share common pathways. In both species, the anther is composed of four distinct somatic cell layers: epidermis, endothecium, middle layer, and tapetum, which surround the developing microsporocytes (Wilson and Zhang, 2009). However, despite the overall similarities, anther developmental steps can be classified as distinct stages when comparing rice and Arabidopsis. The anther development can be mainly categorized into 14 stages and the schematic representation of each can be found in Figure 1. Stages 1 and 2 exhibit analogous major events and morphological markers in both species. Stage 1 starts with the emergence of rounded stamen primordia, consisting of L1, L2, and L3 cellular layers. The L1 cell layer will generate the epidermis, while the L3 cell layer will develop into vascular and connective tissues (Goldberg et al., 1993). In stage 2, the L2 cell layer will give rise to the archesporial cells, while the stamen primordia differentiate into round-shaped structures. In stage 3, by rice classification, periclinal divisions of archesporial cells generate primary parietal cells. In Arabidopsis, the four regions start the mitotic activity of archesporial cells which derive primary parietal cells and sporogenous cells, and further divisions generate secondary parietal layers and secondary sporogenous cells (McCormick, 1993; Zhang *et al*., 2011). In rice, the emergence of the primary sporogenous cells occurs exclusively in stage 4, coinciding with the development of two secondary parietal layers formed by the primary parietal cells. Moreover, in rice and Arabidopsis, during stage 4, the anther undergoes a transformation into a four-lobed structure showing the growth of two stomium regions, accompanied by the initiation of the vascular region. In rice, at stage 5, primary sporogenous cells divide to form secondary sporogenous cells. Simultaneously, the outer secondary parietal layer develops into the endothecium and middle layers, while the inner secondary parietal layer differentiates into the tapetum. During stage 5, in Arabidopsis, the anther exhibits four well-defined locules, each presenting all the different anther cell types. During this stage, microspore mother cells (MMC) originate from secondary sporogenous cells. Conversely, in rice at stage 6, the secondary sporogenous cells generate the MMC. In Arabidopsis, during this same stage, MMC develop within four-layered anther walls and enter meiosis, while the tapetum cells become vacuolated (Scott et al., 2004). Moving to stage 7, in rice, meiocytes initiate meiotic division and are closely associated with the tapetal layer. In Arabidopsis the meiosis culminates with the formation of tetrads. Also, at this stage, the tapetum becomes vacuolated, initiating programmed cell death (PCD). In both rice and Arabidopsis, the middle layer becomes less prominent, with only remnants of the middle layer present at stage 7. In rice, stage 8 is further divided into two sequential steps, 8a and 8b. In 8a, dyads are formed, resulting in one meiocyte containing two nuclei by the end of meiosis I. At this point, the cytoplasm of tapetal cells becomes condensed, initiating PCD (Chen et al., 2005; Li et al., 2006, 2011). In 8b, the tetrads containing microspores are formed after meiosis II, enclosed by the callose wall. The tapetum becomes more condensed and vacuolated. In contrast, in Arabidopsis during stage 8, the callose wall surrounding tetrads degenerates, leading to the release of individual microspores. In rice, during stage 9, the haploid microspores develop an exine wall and are released from the tetrads. At the same time, the tapetal cells undergo condensation, giving rise to distinctive and observable orbicules/Ubisch bodies (Huysmans et al., 1998). In Arabidopsis, the microspores also generate an exine wall and become vacuolated. At these final steps, rice and Arabidopsis once again exhibit identical major events and morphological markers. In stage 10, the tapetum degeneration coincides with the ongoing vacuolation of microspores, which take on a spherical shape. In stage 11, the microspore undergoes its first mitotic division, producing generative and vegetative cells. Tapetum cells almost entirely degenerate into cellular debris and Ubisch bodies (Li and Zhang, 2010). In stage 12, the generative cell undergoes the second mitosis, forming tricellular pollen grains. The tapetum completely disappears at this stage. Finally, in stage 13/14, the flower opens, and anther dehiscence occurs, enabling the release of the mature pollen grains, respectively. For a complete and detailed description of all stages of anther development, readers can consult reviews by Sanders et al. (1999), Itoh et al. (2005), and Zhang *et al* (2011).

Structures such as tapetum and pollen wall are essential for the development and protection of pollen grains. Throughout anther development, the tapetum plays a crucial role as a critical layer of cells supporting the development of microspores into mature pollen grains. This includes the synthesis of lipids and proteins that form the pollen coat in the later stages (Ma et al., 2021). The tapetum is derived from the innermost layer of the anther wall, surrounding the developing microspores providing them lipids, carbohydrates, and proteins. The tapetum undergoes PCD, which initiates at stage 8a in rice, and stage 7 in Arabidopsis, and continues until completely disappears at stage 12, allowing other substances to be released and incorporated into the developing pollen grains (Goldberg et al., 1993; Zhang et al., 2011). 

The multilayered pollen wall, which envelops the pollen grains, serves as a specialized cell wall that not only offers a mechanical safeguard to male gametophytes against desiccation, environmental stressors, and microbial assaults, but also plays a crucial role in diverse aspects of pollination, including pollen adhesion, hydration, and germination (Dickinson and Lewis, 1973; Scott et al., 2004). The outer layer of the pollen wall, known as exine, primarily consists of sporopollenin, a highly durable biopolymer derived from fatty acids, phenolics, and trace amounts of carotenoids (Ahlers et al., 1999). Sporopollenin is considered one of the most resilient biopolymers due to its remarkable tolerance to desiccation and various stresses and its insolubility in strong acids, bases, and oxidizers (Xu et al., 2014). In Arabidopsis and other plants, the pollen coat is also attached to the pollen exine and presents compounds important for fertilization signals (Ma et al., 2021). The intine is the innermost layer of the pollen wall, which is composed of cellulose, hemicellulose, and pectin. It is crucial in protecting the genetic material during pollen development and transport. The intine layer is responsible for maintaining the pollen grain’s structural integrity and regulating water uptake and release during pollen hydration and dehydration (Pacini and Franchi, 1992).

## bHLH transcriptional factor family

The basic helix-loop-helix (bHLH) transcription factor family is a large group of proteins that play crucial roles in regulating gene expression and controlling various cellular processes in eukaryotes. In plants the bHLH transcription factor family is a diverse and essential group of proteins acting as transcriptional regulators in different aspects of plant growth, development, and stress responses. In rice (*Oryza sativa* L.) there are 177 members of this family, while Arabidopsis has 162 members, making it the second-largest family of transcription factors in plants (Heim et al., 2003; Li et al., 2006; Babitha et al., 2015; Xu et al., 2015; Qian et al., 2021).

bHLH proteins are characterized by a conserved structural motif consisting of a basic DNA-binding domain and a helix-loop-helix dimerization domain. The basic DNA-binding domain allows bHLH proteins to recognize and bind to specific DNA sequences in the promoter regions of target genes, while the helix-loop-helix dimerization domain enables bHLH proteins to form homodimers or heterodimers with other bHLH proteins, leading to the formation of transcriptional complexes (Atchley et al., 1999).

The basic region of bHLH proteins, which contains around 15 amino acids with six basic residues, is highly conserved and encompasses the HER motif (His5-Glu9-Arg13) (Atchley et al., 1999; Toledo-Ortiz et al., 2003). Through the basic region, bHLH proteins bind to the E-box (5’-CANNTG-3’) present in the promoter of genes involved in various metabolic pathways. Notably, a specific member of the E-box family, known as the G-box (5’-CACGTG-3’), can be recognized by approximately 81% of bHLHs (Qian et al., 2021). The HLH region, located in the carboxy-terminal portion, consists of approximately 40-50 amino acids arranged in two amphipathic helices with hydrophobic residues linked by a variable-length loop (Nair and Burley, 2000). Some proteins within the bHLH family have lost their basic domain and are called HLH proteins. These HLH proteins act as negative regulators, forming heterodimers that cannot bind to DNA (Fairman et al., 1993).

## Anther developmental genes regulated by bHLHs

Transcriptomic analyses of rice anther development have revealed that numerous genes involved in tapetum PCD, lipid exine formation, and other key processes during the final anther development stages are direct or indirect targets of bHLH (Huang et al., 2009). Among these genes, aspartic proteases, including OsAP25 and OsAP37, are key initiators of PCD in plants and are involved in tapetal PCD initiation (Chen et al., 2009). The ANTHER DEVELOPMENT F-BOX (OsADF), a panicle-specific F-box protein, significantly impacts pollen development by contributing to tapetal PCD (Li et al., 2015). Furthermore, cysteine proteases (CPs), which constitute a group of enzymes intricately involved in intracellular protein degradation and in the process of PCD, serve to underscore the crucial role of the tapetum in anther development (Solomon et al., 1999). *OsCP1*, a gene encoding a cysteine protease in rice, stands out as particularly significant, as mutations in this gene disintegrate microspores after their release from tetrads (Lee et al., 2004).

Sporopollenin precursors primarily consist of complex biopolymers derived from saturated compounds like long-chain fatty acids and aliphatic chains. A variety of enzymes directly involved in this biosynthesis process are transcriptionally regulated by the bHLH TF family, including DEFECTIVE POLLEN WALL (DPW), POLYKETIDE SYNTHASE (PKS), and cytochrome P450 family members. *OsDPW* encodes a fatty acyl carrier protein reductase, which is essential for anther cuticle and pollen sporopollenin biosynthesis (Shi et al., 2011). OsPKS1 and OsPKS2 play roles in condensing fatty acyl-CoA into components of the sporopollenin precursor (Shi *et al*., 2018; Zou et al., 2018). The cytochrome P450 family members, OsCYP703A3 and OsCYP704B2, are responsible for fatty acid hydroxylation, contributing to cutin and sporopollenin biosynthesis (Li et al., 2010; Yang et al., 2014). Additionally, lipid transfer proteins (LTP), such as OsC6 (LTPL68), OsC4 (LTP44), and OsLTPL94, are crucial for rice pollen wall development. While OsC6 is widely distributed in anther tissues, OsC4 appears specific to the tapetum, facilitating the transfer of lipid molecules from metabolically active tapetal cells to other anther cells for orbicule and pollen wall development (Tsuchiya et al., 1992; Zhang D et al., 2010). OsLTPL94, a non-specific lipid transfer protein, is likely secreted by both pollen mother cells and the tapetum, ensuring the proper assembly of sporopollenin for microspore exine development (Tao et al., 2021). In parallel, ABC TRANSPORTER G FAMILY MEMBERS, including OsABCG15 and OsABCG26, are situated in the tapetum membrane forming homo/hetero-dimers. These dimerized OsABCG proteins play a pivotal role in transporting the synthesized lipid precursors from the tapetal interior to the exterior. Similarly, OsC6 and OsC4 proteins secreted from tapetum cells transport lipid precursors to the surfaces of epidermis and microspores, contributing to cuticle and exine development (Qin et al., 2013; Wu et al., 2014; Zhao et al., 2015). 

## bHLHs involved in anther development

bHLH transcriptional factors regulate genes that control cell differentiation and division and genes that are important for forming the cell walls surrounding the developing pollen grains. Besides, bHLH factors also control the expression of genes involved in hormone synthesis, including gibberellins and cytokinins, which are essential for anther development (Heim et al., 2003; Plackett et al., 2011; Reyes-Olalde et al., 2017). In the subsequent sections, we explore the key bHLH transcription factors involved in rice anther development and their orthologs in *Arabidopsis thaliana*. In addition, all bHLHs cited in this section are summarized in Table 1. These transcription factors control anther morphology, meiotic development, and tapetum degeneration. As described in detail in the following sections, misregulation of the expression of these bHLH factors can result in significant defects such as morphological abnormalities of the anther, inability to complete meiotic cytokinesis, the collapse of microspores, and male sterility.

**Table 1 -:** bHLHs involved in anther development

Gene	Locus	Mutant phenotype	References
UDT1, *UNDEVELOPED TAPETUM 1* (*OsbHLH164*)	LOC_Os07g36460	The *udt1* mutant resulted in male sterility due to abnormal tapetal development and inhibited meiocyte development	Jung et al., 2005.
DYT1, *DYSFUNCTIONAL TAPETUM 1*	At4g21330	The *dyt* mutant shows abnormal anther morphology, and meiocytes can complete meiosis I but do not form a thick callose wall, frequently fail to complete meiotic cytokinesis, and eventually collapse.	Cui et al., 2016. Zhang et al., 2006.
TDR, *TAPETUM DEGENERATION RETARDATION* (*OsbHLH005*)	LOC_Os02g02820	The *tdr* mutant is male sterile and shows delayed degeneration of the tapetum and middle layer, along with the collapse of microspores.	Li et al., 2006.
AMS, *ABORTED MICROSPORE*	At2g16910	The *ams* mutants displayed impaired release of microspores, a deficiency in sporopollenin deposition, and a substantial decrease in total phenolic compounds and cutin monomers.	Xu et al., 2014. Sorensen et al., 2003.
TIP2, *TDR INTERACTING PROTEIN 2* (*OsbHLH142*)	LOC_Os01g18870	The *tip2* mutants manifest complete male sterility as they exhibit undifferentiated inner three layers of anther wall and fail to undergo tapetal programmed cell death.	Ko et al., 2017. Fu et al., 2014.
bHLH010 bHLH089 bHLH091	At2g31220 At1g06170 At2g31210	The combinations of double and triple mutations exhibit strong anther phenotypes such as abnormal tapetum morphology, delayed callose degeneration, and aborted pollen development.	Zhu et al., 2015.
EAT1, *ETERNAL TAPETUM 1* (*OsbHLH141*)	LOC_Os04g51070	The *eat1* mutant displays delayed meiosis with abnormally decondensed chromosomes, leading to the formation of abortive microspores due to abnormal programmed cell death of the tapetum.	Niu et al., 2013.
*OsbHLH035*	LOC_Os01g06640	*OsbHLH035* overexpression plants displayed smaller and curved anthers. The anthers exhibited abnormal changes in the endothecium, sterile pollen sacs, and pollen grains with atypical vacuolation, as well as changes in size.	Ortolan et al., 2021.

### *UDT1* and *DYT1* are essential for anther development in rice and Arabidopsis, respectively 

The *UNDEVELOPED TAPETUM1* (*UDT1*, LOC_Os07g36460) encodes a crucial bHLH transcription factor responsible for tapetal cell maturation and the differentiation of secondary parietal cells in rice. As reported by Jung et al. (2005), *UDT1* exhibits a preferential expression pattern during the early stages of anther development, with peak expression between stages 6 and 8b (Figure 1). During this period, tapetal cells demonstrate the highest transcriptional activity among all anther cell types. *UDT1* expression is detected in both the anther wall and meiocytes but declines in the later stages (stages 9-14), implying that UDT1 not only initiates cellular differentiation but also sustains anther formation. Disruptions in the *UDT1* gene result in male sterility, with mutant anthers lacking mature pollen grains and failing to produce fertile seeds, highlighting the critical role of UDT1 in tapetum development from the early meiosis stage (Jung *et al.*, 2005). Notably, during the pre-meiosis stage, *udt1* mutant anthers display normal development of primary sporogenous cells and the four anther wall layers. However, as meiosis begins (stages 7-8a), the tapetal layers in the mutant anthers undergo premature degeneration and dyads fail to develop into tetrads. By late meiosis (stage 8b), *udt1* mutant meiocytes experience severe contractions, accompanied by numerous small vacuoles, ultimately resulting in the presence of only remnants of meiocytes in the mutant locules. These detrimental effects can be attributed to the absence of *UDT1* gene function, as its transcript is predominantly found in all cell types within early anthers (Jung *et al*., 2005).

**Figure 2 -:**
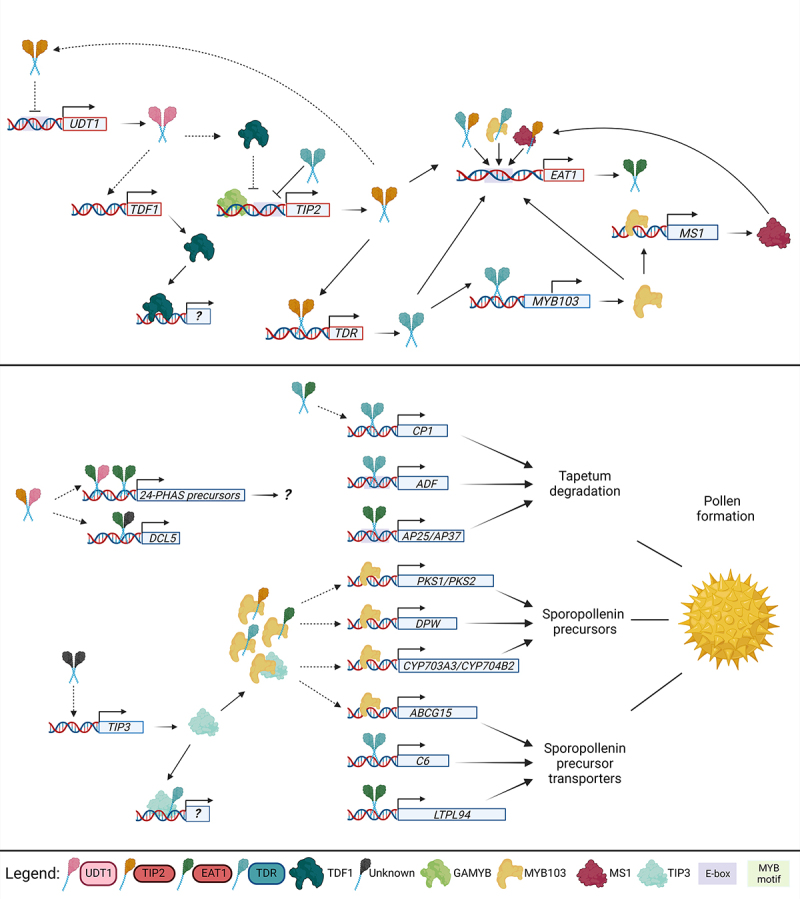
Proposed model for bHLH regulatory pathway of anther development in rice. The top panel illustrates the regulatory network primarily active during the meiotic phase of anther development, with a predominant role of bHLH transcription factors. In contrast, the bottom panel depicts the regulatory pathway during the microspore maturation phase, highlighting genes directly associated with tapetum programmed cell death (PCD) and pollen wall formation. Lines indicate direct regulation, while dashed lines indicate possible regulation. Arrows indicate induction while dashes indicate repression. Taper lines indicate the product of the expression. The color of the boxes in the legend, as well as the color and shape of the protein are the same between the orthologs in Arabidopsis in Figure 3. UDT1: UNDEVELOPED TAPETUM1; TDF1: DEFECTIVE IN TAPETAL DEVELOPMENT AND FUNCTION1; TIP2: bHLH142/TDR INTERACTING PROTEIN2; GAMYB: GIBBERELLIN MYB GENE; TDR: TAPETUM DEGENERATION RETARDATION; EAT1: ETERNAL TAPETUM1; OsCP1: CYSTEINE PROTEASE1; OsADF: ANTHER DEVELOPMENT F-BOX; OsC6: CYSTEINE PROTEASE6; TIP3: TDR INTERACTING PROTEIN3; OsMS1: MALE STERILITY1; OsCYP703A3/OsCYP704B2: CYTOCHROME P450 MEMBERS; OsPKS1: POLYKETIDE SYNTHASE1; OsPKS2: POLYKETIDE SYNTHASE2; OsDPW: DEFECTIVE POLLEN WALL; OsABCG15: ABC TRANSPORTER G15; OsAP25: ASPARTIC PROTEASE25; OsAP37: ASPARTIC PROTEASE37; OsLTPL94: NON-SPECIFIC LIPID TRANSFER PROTEIN94; 24-PHAS: 24-NUCLEOTIDE PHASED SECONDARY SMALL INTERFERING RNAS; DCL5: DICER-LIKE5.

Microarray analysis of *udt1* mutant anthers, as reported by Jung et al. (2005), identified 1225 genes exhibiting significant upregulation or downregulation. Furthermore, several studies revealed the role of UDT1 as a regulator, controlling the expression of numerous genes critical for pollen wall formation and tapetal PCD. In the context of pollen wall formation, UDT1 positively regulates the expression of *OsC6*, *OsC4,* and *OsLTP45*, which encode protease inhibitors and tapetum-specific lipid transfer proteins, respectively (Tsuchiya et al., 1992; Lee et al., 2004; Zhang D et al., 2010; Moon et al., 2020). Additionally, it induces the expression of *OsCYP703A3* and *OsCYP704B2*, genes encoding enzymes critical for anther cuticle and pollen exine formation (Li et al., 2010; Yang et al., 2014; Moon *et al*., 2020). Regarding its role in tapetal PCD, UDT1 serves as a positive regulator for key aspartic proteases including *OsAP37*, *OsAP67*, *OsAP38*, and LOC_Os08g10730 (Chen et al., 2009; Moon *et al*., 2020). Interestingly, a study conducted by Moon *et al*. (2020) revealed significant suppression of *TDR* and *EAT1* transcripts in *udt1* mutants when compared to wild-type plants. However, the reduction in *TIP2* expression remained relatively mild, and it has been shown that UDT1 is unable to bind to the *TIP2* promoter (Ko et al., 2021). Nevertheless, a recent research suggests that most of the downstream candidate genes identified in prior transcriptome analyses may not be immediate downstream targets of UDT1 (Moon *et al*., 2020). Additionally, UDT1 plays a role in the production and processing of 24-PHAS precursors (Ono et al., 2018) (Figure 2). Overall, UDT1 emerges as a crucial element in the complex regulatory pathways governing anther development. Further research is essential to obtain a comprehensive understanding of its specific functions, potential interactions, and direct downstream targets.

In Arabidopsis, the ortholog of *UDT1*, known as *DYSFUNCTIONAL TAPETUM1* (*DYT1*, AT4G21330), is also essential for anther development. The *dyt1* mutant exhibits an aberrant tapetum characterized by premature vacuolation, leading to a failure of microsporocytes to generate microspores following meiotic nuclear division, ultimately resulting in the absence of pollen grains (Zhang et al., 2006). *DYT1* is initially expressed at stage 4 with a drastic reduction at stage 6 (Figure 1), and alongside other bHLHs, forms a feedforward loop that coordinates the anther transcriptional network (Zhu et al., 2011; Cui et al., 2016). DYT1 assumes a pivotal role upstream of over 20 transcription factor genes, notably *AMS*, *MS1*, *TDF1/MYB35*, and *MYB103*. This regulatory effect extends to more than 1000 genes, encompassing functions related to peptide transport, lipid transport, pollen exine formation, pollen development, and phenylpropanoid biosynthesis. Consequently, DYT1 emerges as a central regulator dictating the Arabidopsis anther transcriptome and orchestrating a complex gene expression network (Zhang *et al*., 2006; Feng et al., 2012; Zhu *et al.*, 2015).

**Figure 3 -:**
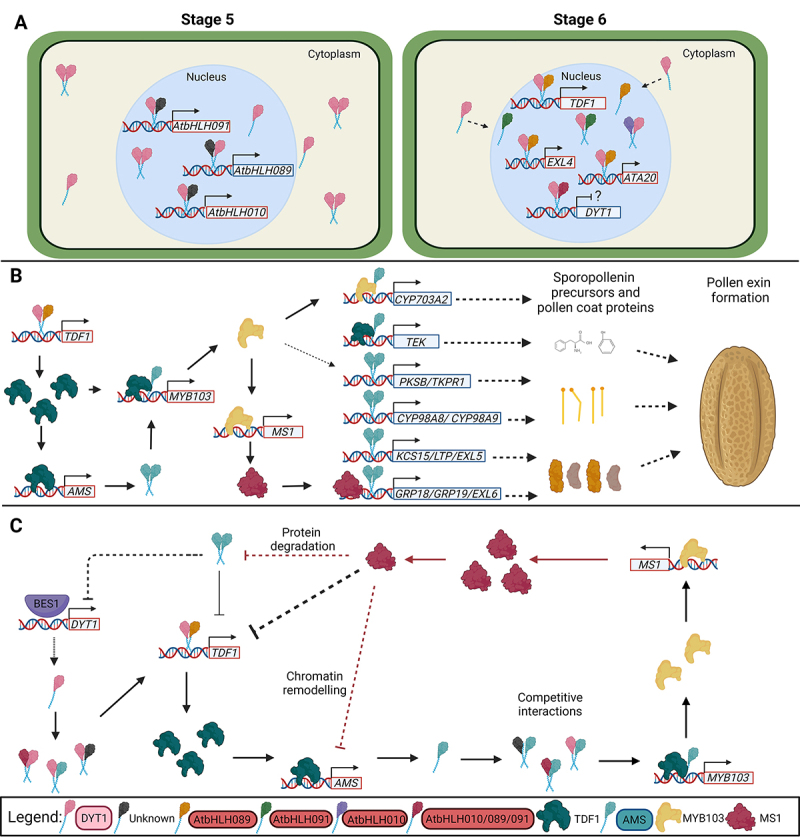
Arabidopsis bHLH involvement in molecular mechanisms of anther development. A) DYT1 is localized in the cytoplasm and nucleus compartments at anther stage 5. It induces the expression of other bHLH genes, whose products interact with DYT1, resulting in DYT1-bHLH010/089/091 heterodimers at anther stage 6. These heterodimers facilitate DYT1 nuclear localization, and their different interaction combinations trigger the transcription of downstream genes, such as *TDF1*, *EXL4,* and *ATA20*. The accumulation of DYT1-bHLH010/089/091 heterodimers suppresses directly or indirectly the expression of DYT1 by negative feedback. B) AMS role during outer pollen layer (exine) wall formation. DYT1 binds to the promoter of the *TDF1* transcription factor and activates its expression. TDF1 activates the expression of downstream genes, such as *AMS*. AMS binds to the promoter of genes related to fatty acid elongation (*KCS15*), fatty acid hydroxylation (*CYP98A8*), lipid metabolism/transport (*LTP*), and production of hydroxylated 𝛂-pyrones (*TKPR1*, *PKSB*), which products are involved in the synthesis of the precursors of sporopollenin biopolymer, to form the exine. Also, AMS potentially activates the expression of pollen coat proteins (*GRP18*, *GRP19*, *EXL5*, *EXL6*) by binding their promoters and can form heterodimers with TDF1 to trigger the expression of *MYB103* and *TEK*. MYB103 directly activates the expression of *MS1*, which also activates the expression of pollen coat proteins. C) Arabidopsis bHLHs feedback regulations. BES1 binds to the *DYT1* promoter and might activate its expression. DYT1 interacts with other bHLHs and regulates several genes, including *TDF1*. TDF1 activates the expression of *AMS*, which product can also interact with many bHLH and regulate a plethora of genes. AMS can promote the expression of *MYB103*, which activates the transcription of *MS1*. MS1 represses the expression of *TDF1* and decreases the AMS protein levels and gene expression, potentially by protein degradation and chromatin remodeling, respectively. AMS interacts with the DYT1 protein and can regulate its expression. The competitive interaction among different bHLHs and transcription factors results in a controlled and coordinated activation/repression of genes involved in anther development and pollen formation. Lines ending with an arrow: direct regulation. Lines ending with a line: repression. Dashed lines: Directly/indirectly or unknown. Red lines: regulation of AMS via MS1. The color of the boxes in the legend, as well as the color and shape of the protein are the same between the orthologs in rice in Figure 2. DYT1: DYSFUNCTIONAL TAPETUM1; EXL4/5/6: EXTRACELLULAR LIPASE 4/5/6; ATA20: ANTHER 20; AMS: ABORTED MICROSPORES; TDF1: DEFECTIVE IN TAPETAL DEVELOPMENT AND FUNCTION 1; BES1: BRI1 EMS SUPPRESSOR 1; KCS15: 3-KETOACYL-COA SYNTHASE 15; CYP98A8/9: CYTOCHROME P450, FAMILY 98, SUBFAMILY A, POLYPEPTIDE 8/9; TKPR1: TETRAKETIDE 𝛂-PYRONE REDUCTASE1; PKSB: POLYKETIDE SYNTHASE B; GRP18/19: GLYCINE-RICH PROTEIN 18/19; MS1: MALE STERILITY1.

Initially located in the cytoplasm, most DYT1 monomers and homodimers are eventually translocated to the nucleus (by an unknown factor) to activate *AtbHLH010/089/091* expression. The interaction between these AtbHLH010/089/091 proteins with DYT1, followed by the formation of heterodimers, enhances the DYT1 nuclear localization and promotes the expression of downstream genes at stage 6 (Figure 3A). Cui et al. (2016) proposed that even at low expression levels during anther stage 5, AtbHLH010/089/091 proteins could interact with DYT1 and translocate it to the nucleus, which would eventually boost their expression at stage 6 (Cui *et al.*, 2016). Additionally, based on the higher gene expression of *DYT1* in the *Atbhlh010/089/091* triple mutant, the accumulation of AtbHLH010/089/091 proteins could negatively regulate the transcription of *DYT1* (Zhu et al., 2015). This exemplifies how different bHLHs can regulate themselves through positive and negative feedback loops to adjust and keep balanced transcript levels necessary for normal anther development.

### TDR and EAT1 regulate tapetum programmed cell death in rice

The rice TAPETUM DEGENERATION RETARDATION (TDR, LOC_Os02g02820) protein is a key player in anther development, primarily governing tapetum PCD. *TDR* is predominantly expressed in tapetal cells, its expression is detected early in anther development, commencing at the meiosis (stage 7) and reaching the maximum level at the young microspore (stage 8b) (Figure 1). However, as anther development progresses to the vacuolated pollen and heading stages, *TDR* transcript levels significantly decrease or become barely detectable (stages 10-14) (Li et al., 2006). The rice *tdr* mutant presents a delay in the tapetum and middle layer degeneration, as well as the collapse of microspores, culminating in complete male sterility. Interestingly, the *tdr* mutant exhibits the normal formation of four anther wall cell layers and the MMC. However, a disruption occurs in post-meiotic development within the tapetum and the middle layer due to the retardation of tapetum PCD (Li *et al.*, 2006).

TDR functions as a transcription factor, actively promoting transcription during the process of tapetal PCD, and it is likely localized within the cell nucleus. It belongs to the MYC (myelocytosis) transcription factor class, characterized by the presence of a basic helix-loop-helix/leucine zipper domain which strongly indicates that TDR is the rice homolog of AMS (Coller et al., 2000; Sorensen et al., 2003; Li et al., 2006). TDR directly regulates the expression of two downstream target genes, *OsCP1* and *OsC6* (Figure 2), genes that encode a cys protease and a protease inhibitor, respectively (Tsuchiya et al., 1992; Solomon et al., 1999; Lee et al., 2004; Zhang D et al., 2010). Additionally, TDR directly and positively regulates *OsADF* binding to the E-box on the *OsADF* promoter (Figure 2) (Li *et al.*, 2015). Other genes are indirectly regulated by TDR, such as *OsMYB103*, *OsPKS1, DPW, CYP703A3, CYP704B2, ABCG15,* and *OsABCG26* (Han et al., 2021; Lei et al., 2022). Unveiling the mechanisms through which TDR influences these genes will significantly enhance our comprehension of TDR’s regulatory functions.

ETERNAL TAPETUM1 (EAT1, LOC_Os04g51070) is a transcription factor that also regulates PCD in tapetal cells during rice anther development acting downstream of TDR (Niu et al., 2013). Its paralog, *OsbHLH142/TIP2*, is found in rice, while three homologs, *AtbHLH010*, *AtbHLH089*, and *AtbHLH091*, are present in Arabidopsis. These genes exhibit an average of 40% identity with *EAT1* in the bHLH domain and the Domain of Unknown Function (DUF) (Niu *et al.*, 2013; Fu *et al.*, 2014; Zhu et al., 2015). The expression of *EAT1* exhibits a bimodal pattern, occurring both during early meiosis (stage 7) and post-meiosis (stages 9-12) (Figure 1). During these stages, it plays a pivotal role in initiating the timely onset of tapetal PCD. In the *eat1* mutant, meiotic division timing is notably delayed, leading to an asynchronous progression within an anther lobe (Ono et al., 2018). The *eat1* mutant also presents delayed tapetal PCD and defective pollen development, displaying abnormal pollen exine patterns, ultimately resulting in complete male sterility (Niu *et al.*, 2013).

Molecular analysis of EAT1’s functions reveals that it regulates genes involved in lipid metabolism and pollen coat formation during meiosis, underpinning its multifaceted role. EAT1 directly regulates the expression of *OsAP25* and *OsAP37*, which encode aspartic proteases responsible for initiating PCD in plants. EAT1 binds to the E-box-containing promoters of *OsAP25* and *OsAP37* to execute its regulatory function (Figure 2) (Chen et al., 2009; Niu et al., 2013). EAT1 also positively regulates *OsLTPL94* by directly binding to its promoter (Figure 2) (Tao et al., 2021). Additionally, EAT1 interacts with UDT1 and promotes the transcription of 24-nucleotide phased secondary small interfering RNAs (phasiRNAs) precursors, influencing 24-nt small RNA production (Figure 2). The temporal shift from UDT1 to TDR binding partners enables EAT1 to modulate downstream targets from meiotic phasiRNA production to postmeiotic tapetal PCD induction (Figure 2) (Ono et al., 2018). Furthermore, EAT1 contributes to the transcription of *DICER-LIKE5* (*DCL5*), a crucial player in the processing of double-stranded 24-PHASs into 24-nt lengths (Figure 2). The expression of EAT1 is influenced in *gamyb* and *udt1* mutants, with *tdr* and *ms1/ptc1 persistent tapetal cell1* mutants presenting a great reduction in its expression, indicating that TDR and MS1/PTC1 play a significant role in positively regulating EAT1 (Ono *et al*., 2018).

### AMS plays a role in tapetal and microspore development in Arabidopsis

The Arabidopsis AMS (ABORTED MICROSPORE, AT2G16910) is a protein that plays a crucial role in tapetal and microspore development within the developing anther. It functions as a transcription factor and it belongs to the MYC class, characterized by the presence of a bHLH domain. AMS is an early-acting regulator of pollen mitosis I, potentially through the relaxation of chromatin structure (Sorensen et al., 2003; Xu et al., 2010). According to Zhu et al. (2011), *AMS* expression is low at stage 5 in wild-type anthers, increasing during meiosis (stage 6), mainly in the tapetal cells (Figure 1). After microspore release (stage 8), the expression of *AMS* is still detectable in the tapetum and microspores. The *ams* mutant shows defective microspore release, a lack of sporopollenin deposition, and a dramatic reduction in total phenolic compounds and cutin monomers. Additionally, the *ams* mutant displays abnormally enlarged tapetal cells and aborted microspore development, with a frequent observation of abnormal tetrads after pollen mother cell meiosis (Sorensen *et al.*, 2003). AMS acts downstream to DYT1, but upstream to many genes related to the synthesis of sporopollenin precursors and it is considered a master regulator of pollen wall architecture (Xu *et al.*, 2014).

###  TIP2 directly activates the expression of *TDR* and *EAT1*

bHLH142/TDR INTERACTING PROTEIN2 (TIP2, LOC_Os01g18870) is a bHLH transcription factor characterized by conserved bHLH and DUF domains. TIP2 controls cell differentiation and morphogenesis in the endothecium, middle layer, and tapetum, playing a crucial role in regulating normal meiosis and the release of microspores from the tetrad. The expression pattern of *TIP2* is highly specific to anther tissues. It initiates at the onset of meiosis (stage 6) and maintains consistent expression throughout mitosis. The highest expression levels are achieved at stages 7 and 8, and this expression level persists up to stage 10 (Figure 1) (Fu et al., 2014; Ko et al., 2014).

Mutations in *TIP2* result in undifferentiated inner three anther wall layers and aborted tapetal PCD, ultimately leading to complete male sterility. The *tip2* mutants exhibit smaller anthers and fail to produce mature pollen grains. These mutants present vacuolated and expanded cells within the three inner layers, coupled with the presence of microspore mother cells that fail to mature into viable pollen grains (Fu et al., 2014; Ko et al., 2014). Furthermore, in *tip2* mutants, there is a substantial reduction in the transcription of genes linked to critical processes such as callose degradation, lipid metabolism, and transport. Among these genes, *OsCYP703A3*, *OsCYP704B2*, *OsC6*, and *OsDPW* stand out with significantly decreased in expression levels (Fu *et al*., 2014). TIP2 plays a pivotal role as an upstream regulator of both *TDR* and *EAT1*, directly influencing their expression. Furthermore, TIP2 can interact with TDR forming a heterodimer that collectively controls the expression of *EAT1* (Figure 2) (Ko *et al.*, 2014; Ko *et al.*, 2017).

In addition, the expression levels of critical transcription factors, including *TDR*, *EAT1*, and *MS1/PTC1*, are evident in the absence of TIP2. Conversely, *UDT1* displays upregulation under these circumstances. This observation suggests that TIP2 likely functions as a positive regulator in governing the expression of *TDR*, *EAT1*, and *MS1/PTC1* within the tapetal cells (Fu et al., 2014). Furthermore, there is a possibility of a feedback regulatory mechanism between TIP2 and UDT1, as supported by the presence of E-box elements in the *UDT1* promoter (Figure 2) (Jung et al., 2005; Fu *et al*., 2014). Thus, TIP2 emerges as a pivotal orchestrator of the differentiation and function of the tapetum and inner anther wall layers, ultimately contributing to the successful development of the anther.

### *AtbHLH010/089/091* are preferentially expressed in the tapetum of the Arabidopsis anther in a DYT1-dependent manner 

The *AtbHLH010/089/091* genes in the Brassicaceae (crucifer) family originated from recent gene duplications in their most recent common ancestor. These three genes, namely *bHLH010* (AT2G31220), *bHLH089* (AT1G06170), and *bHLH091* (AT2G31210) share similar sequences and expression patterns, indicating potential overlapping or redundant functions. They encode proteins with strong nuclear localization signals and are preferentially expressed in the tapetum of the Arabidopsis anther in a DYT1-dependent manner (Zhu et al., 2015). At stage 5, *bHLH010*, *bHLH089*, and *bHLH091* show weak expression in the tapetum and microsporocytes, which increased at stage 6 and peaked at stage 7 in both the tapetal layer and microsporocytes (Figure 1) (Zhu *et al*., 2015). While single mutants of these genes do not exhibit developmental abnormalities, various double and triple combinations progressively resulted in increasingly defective anther phenotypes, such as abnormal tapetum morphology, delayed callose degeneration, and aborted pollen development, indicating their redundant functions in male fertility. The triple mutant exhibited severely defective anther phenotypes, similar to the *dyt1* mutant phenotype, resulting in complete seed sterility (Zhu *et al.*, 2015). Expression analysis revealed that the genes involved in pollen formation, such as *MS2* (*MALE STERILITY 2*), *MEE48* (*MATERNAL EFFECT EMBRYO ARREST 48*), and *LAP6* (*LESS ADHESIVE POLLEN 6*), are altered in both the bHLH triple mutant and *dyt1* mutant. However, *LAP5* (*LESS ADHESIVE POLLEN 5*) and *ACOS5* (*ACYL-COA SYNTHETASE 5*) are significantly affected only in the *dyt1* mutant and not in the bHLH triple mutant (Zhu *et al.*, 2015).

Recently, analyses in the *bhlh010 bhlh089* double mutant exhibited defective pollen exine and intine development. Moreover, metabolomic and transcriptomic analyses suggested that bHLH010 and bHLH089 regulate different metabolic pathways, such as fatty acid biosynthesis, sugar metabolism, flavonols, cellulose synthesis, and transport of metabolites, suggesting they might regulate both metabolite synthesis and transport, thereby playing a role in the pollen exine and intine development (Lai et al., 2022). Despite potentially regulating the expression of the same set of genes, efforts are being made to identify their functional differences (Fu et al., 2020; Lai *et al.*, 2022). For instance, bHLH010 and bHLH089 share the ability to activate the expression of *CSLD5* (*CELLULOSE SYNTHASE-LIKE D5*), *CSLD6* (*CELLULOSE SYNTHASE-LIKE D6*), *LAP6*, and *UGT85A5* (*UDP-GLUCOSYL TRANSFERASE 85A5*) genes, but *FRA8* (*FRAGILE FIBER 8*) and *TSM1* (*TAPETUM-SPECIFIC METHYLTRANSFERASE 1*) are specifically induced by bHLH089, and *CSLB03* (*CELLULOSE SYNTHASE-LIKE B3*) and *SUS3* (S*UCROSE SYNTHASE 3*) are specifically induced by bHLH010 (Lai *et al.*, 2022). Most of these genes are related to pollen development, although the role of some of them, such as *CSLD5/6* and *UGT85A5*, is still unknown in this process. Furthermore, the heterodimer of bHLHL089 and DYT1 can bind to an E-box variant promoter and activate the expression of other anther development genes, such as *ATA20* (*ANTHER 20*), *EXL4* (*EXTRACELLULAR LIPASE 4*), *MEE48,* and *MYB35* (*MYB DOMAIN PROTEIN 35*) (Cui et al., 2016; Fu *et al.*, 2020).

### *OsbHLH035*: another family member involved in anther formation 

OsbHLH035 (LOC_Os01g06640) was recently identified as an important regulator of anther development in rice. Its expression is specific to the MMC formation (stage 6) of anther development and is also found in flower primordia and young palea and lemma (Figure 1) (Ortolan et al., 2021). Proper anther maturation seems to require precise regulation of *OsbHLH035* expression. Sustained expression of this gene through maize *ubiquitin1* promoter results in plants with small and curved anthers and a reduction of 72% in seed production. Pollen grains from transgenic plants displayed various cytological alterations, such as atypical vacuolation, cytoplasm with pyknotic material, loss of cytoplasm and nuclear content, the collapse of the entire grain, and size alterations. Transgenic anthers presented significant modifications in the subdermal layers, mainly in the endothecium (Ortolan *et al.*, 2021). Three members of the GRF (Growth Regulating Factor) family, OsGRF3, OsGRF4, and OsGRF11, were identified as direct regulators of *OsbHLH035* expression through yeast one-hybrid. By transactivation assays, it was confirmed the direct negative regulation of *OsbHLH035* by OsGRF11 (Ortolan *et al.*, 2021). Despite the importance of OsbHLH035’s regulatory role in microsporangia development, there is a lack of clarity regarding its specific targets and potential interacting partners. Therefore, further investigations are warranted to elucidate its precise function and its position within the regulatory pathway of anther development.

## Regulatory networks involving bHLHs and other transcription factors

Anther development is governed by a sophisticated regulatory network. The meiotic phase, encompassing stages 6-9 in rice and stages 5-9 in Arabidopsis, relies significantly on the involvement of bHLH TFs as key regulators. However, it is imperative to acknowledge that these stages encompass several genes that play indispensable roles, either by modulating gene expression or by interacting with the bHLH TFs. In the ensuing discussion, we explore the progression of this intricate regulatory network, which is structured to correspond with the various distinct stages of anther development. This comprehensive discussion includes not only the previously mentioned bHLH TFs but also introduces other pivotal players that are critical to this elaborate process.

The transcription factor GIBBERELLIN MYB GENE (GAMYB, LOC_Os01g59660) is implicated in rice anther tapetal and pollen development. Ko et al. (2021) demonstrated that GAMYB plays a crucial role in modulating the expression of *OsbHLH142/TIP2* during the early stages of pollen development. Specifically, GAMYB binds to the MYB motif on the *TIP2* promoter, while TDR acts as a transcriptional repressor of this regulation by binding to the E-box element near the MYB motif (Figure 2). The expression of *GAMYB* is predominantly observed during the meiosis and young microspore (stages 6-8b) (Figure 1). Furthermore, GAMYB likely operates in parallel with UDT1, influencing anther development and the regulatory hierarchy of OsbHLH142/TIP2, which is positioned downstream of GAMYB- and UDT1-dependent pathways, acting as a central hub in the intricate network of rice pollen development regulation (Fu *et al*., 2014).

*DEFECTIVE in TAPETAL DEVELOPMENT and FUNCTION1* (*OsTDF1*) is the rice ortholog of Arabidopsis TDF1, encoding a protein with an R2R3 domain from the MYB superfamily. The *OsTDF1* transcript was predominantly detected in tapetal cells from the onset of meiosis and at the tetrad, stages 6-8b. The *OsTDF1* expression decreased in the tapetum after microspore release (stage 9) (Cai et al., 2015). The *ostdf1* mutant presents enlarged, vacuolated tapetal cells that fill the locular space, ultimately crushing the unreleased tetrads. The knockout of *OsTDF1* severely impairs tapetum development and leads to the failure of middle-layer cell degeneration in rice, ultimately resulting in male sterility (Cai *et al*., 2015). Moreover, genes such as *OsAP19*, *OsAP25*, *OsAP37*, and *OsCP1* were downregulated in *ostdf1* inflorescences. Interestingly, the expression levels of *TDR* and *EAT1* were found to be reduced by approximately 60% in the *ostdf1* mutant, simultaneously repressing the expression of *OsMYB103* and *MS1/PTC1* (Cai *et al.* 2015). This observation strongly suggests that OsTDF1 assumes a critical role as a regulator of tapetum PCD, middle-layer degeneration, and pollen wall, primarily through the regulation of TDR and EAT1, and consequently, their downstream targets. Surprisingly, the expression of *OsbHLH142/TIP2*, a transcription factor known to interact with TDR, was upregulated in *ostdf1*, adding a layer of complexity to the regulatory network involving OsTDF1 (Figure 2) (Cai *et al*., 2015). However, further investigations are needed to ascertain whether TDR and EAT1 are direct targets of TDF1, as well as to explore the potential existence of regulatory feedback between TDF1 and TIP2.

The PHD-finger proteins MALE STERILITY1 (MS1, LOC_Os09g27620) and TDR INTERACTING PROTEIN3 (TIP3, LOC_Os03g50780) were identified as transcriptional activators involved in tapetum PCD and pollen wall construction (Yang et al., 2019a, b). *OsMS1*, also referred to as *PERSISTENT TAPETAL CELL1* (*PTC1*), is orthologous to Arabidopsis *MS1* (Li et al., 2011; Yang *et al*., 2019a). *OsMS1* is mainly expressed in anthers between stages 8b and 14, with higher expression observed at stage 9 (Figure 1) (Yang *et al*., 2019a). The anthers of the *ms1* mutant appeared slightly yellow and smaller, displaying complete male sterility without mature pollen grains. Furthermore, in the *ms1* mutant the expression of *EAT1*, *OsAP37*, *OsAP25*, *OsC6* and *OsC4*, were significantly reduced. Yang *et al*. (2019a) also demonstrated the interaction between OsMS1 and TIP2 through which they regulate the expression of *EAT1* (Figure 2). This interaction includes OsMS1 as a part of the regulation network of tapetum development and PCD in rice, but further analyses are necessary to confirm its direct targets.

TIP3 regulates Ubisch body morphogenesis and pollen wall formation. Its expression was initially detected mainly in anther somatic layers at stage 6, and then the strong expression signal was detected predominantly in the tapetum and microspores from stage 8a to 10 (Figure 1) (Yang et al., 2019b). The anthers of the *tip3* mutant are shorter, pale yellow, and lack visible pollen grains, resulting in complete male sterile plants. The *tip3* mutants also present changes in the expression of several genes such as *OsCP1*, *OsAP25*, *OsAP37*, *OsDPW*, *OsCYP703A3*, *OsCYP704B2*, *OsC6*, *OsABCG15* and *OsABCG26*. Furthermore, it was demonstrated that TIP3 interacts with TDR, suggesting a key role in regulating tapetum development and pollen wall formation (Figure 2) (Yang *et al*., 2019b). However, further studies are needed to identify genes directly regulated by TIP3, and whether this regulation is dependent on the interaction with TDR.

Another MYB TF, OsMYB103 (LOC_Os04g39470) (also referred to as BM1, MS188, and MYB80) plays a pivotal role in the complex regulatory network governing anther development. *OsMYB103* is an ortholog of Arabidopsis AtMYB103 and encodes an R2R3 MYB transcription factor (Zhang S et al., 2010). This gene has been the focus of multiple independent studies, which have revealed some discrepancies while simultaneously validating specific similarities in the results. There is a contradiction between gene expression in the anther development stages, nonetheless, Han et al. (2021) showed that *OsMYB103* transcripts gradually increased in tapetal and meiocyte cells by stage 7, and the highest level was observed in tapetal cells at the tetrad stage (8b) (Figure 1). All mutants analyzed are male-sterile, presenting delayed tapetum degradation and defective pollen, with anthers presenting slight withering, aberrant vacuolized tapetal cells, absence of a sexine layer, and defective anther cuticle (Han *et al*., 2021). Concerning protein dimerization, Xiang et al. (2021) demonstrated that OsMYB103 physically interacts with bHLHs TIP2, EAT1, and PHD (plant homeodomain)-finger member, TIP3. While other studies have shown that OsMYB103 and TDR can interact with each other, and *OsMYB103* expression is directly regulated by TDR (Figure 2) (Han *et al*., 2021; Lei et al., 2022). Han *et al*. (2021) also showed that OsMYB103 directly regulates the expression of multiple genes involved with sporopollenin synthesis and transport including *OsCYP703A3* and *OsCYP704B2*, *OsPKS1* and *OsPKS2*, *OsDPW*, and *OsABCG15* (Figure 2). OsMYB103 also directly regulates the expression of *EAT1* and *OsMS1*, which positively regulate tapetum degradation (Figure 2) (Lei *et al*., 2022). Finally, both studies highlighted OsMYB103 as a fundamental component of rice anther development with a key role in tapetal and microspore development (Han *et al*., 2021; Xiang *et al*., 2021; Lei *et al*., 2022).

The 24-nucleotide phased secondary small interfering RNAs (phasiRNAs) are a unique class of plant small RNAs abundantly expressed in monocot anthers at early meiosis. It has recently emerged as playing an important role in monocot anthers; they also play a crucial role in maintaining genome integrity by suppressing the activity of transposable elements (Ono et al., 2018). Specifically, various bHLH proteins such as TIP2 and UDT1 are involved in meiotic small RNA biogenesis in the anther tapetum. Studies also highlight EAT1 as a key regulator responsible for triggering meiotic phasiRNA biogenesis in the anther tapetum (Figure 2). TIP2 potentially interacts with both UDT1 and TDR, indicating its involvement in activating the transcription of 24-PHASs and *DCL5* during early meiosis (Figure 2) (Ono *et al*., 2018). These findings provide valuable insights into the complex regulatory network involved in anther development during early meiosis.

Finally, we present a comprehensive model for the bHLH regulatory pathway of anther development in rice, illustrated in Figure 2. Starting with two key transcription factors, UDT1 and TDF1, which are positioned upstream of various genes. Although no direct targets of UDT1 and TDF1 have been previously identified, given the broad range of genes they regulate it is presumed that UDT1 directly regulates *TDF1*. Interestingly, both UDT1 and TDF1 appear to exert a negative regulatory effect on *TIP2* expression. UDT1 does not bind to the *TIP2* promoter, raising the question of whether TDF1 negatively regulates *TIP2* by directly binding to its promoter. TIP2 plays a pivotal role in this regulatory network, and its expression is intricately controlled. There is a mechanism in which GAMYB induces *TIP2* expression by binding to the MYB motif on its promoter. In contrast, TDR acts as a repressor by competing with GAMYB, binding to the E-box on the *TIP2* promoter. TIP2 has a direct role in regulating *TDR* expression and interacts with TDR to induce *EAT1* expression. Additionally, TIP2 appears to act as a negative regulator of *UDT1*, suggesting a possible feedback regulatory loop between TIP2 and UDT1. TDR takes an upstream position in regulating several genes and directly regulates the expression of *OsMYB103*, *OsCP1*, *OsADF*, and *OsC6*. OsMYB103 interacts with TDR to induce *EAT1* expression and physically interacts with TIP2, EAT1, and TIP3. OsMYB103 is a direct regulator of several genes, including *OsMS1*, *OsCYP703A3*, *OsCYP704B2*, *OsPKS1*, *OsPKS2*, *OsDPW*, and *OsABCG15*. However, it is not fully clear which partners of OsMYB103 are involved in the regulation of each of these genes. EAT1 directly regulates the expression of *OsAP25* and *OsAP37* as well as *OsLTPL94*. MS1 plays a positive regulatory role for several genes and can interact with TIP2 to enhance the expression of *EAT1*. TIP3 is responsible for the regulation of several genes and can interact with TDR. As for both TIP3 and MS1, their direct targets remain unidentified. The specific function of 24-PHAS in anther development is not yet clear, but their involvement is essential for proper anther development. The 24-PHAS precursors are directly regulated by the EAT1-UDT1 dimer, and it seems that the TIP2-UDT1 dimer is also involved in this regulation. Furthermore, EAT1, in conjunction with an unknown partner, contributes to the transcription of *DCL5*. 

In Arabidopsis, DYT1 is considered a crucial transcriptional regulator in the early stages of tapetal development following the initiation of anther cell layers. Previous studies positioned DYT1 downstream SPL/NZZ (SPOROCYTELESS/NOZZLE) and EMS1/EXS (EXCESS MICROSPOROCYTES 1/EXTRA SPOROGENOUS) and upstream TDF1, AMS, and MS1 (MALE STERILITY 1, AT5G22260) in the regulatory hierarchy (Zhang et al., 2006; Zhu et al., 2008). It was shown that BRI1 EMS SUPPRESSOR 1 (BES1) and BRASSINAZOLE RESISTANT 1 (BZR1) not only participate in the brassinosteroid signaling pathway, but they can also bind to the *DYT1* promoter and, therefore, might regulate its activity (Chen et al., 2019). In a yeast-two hybrid assay, DYT1 was found to form homodimers, as well as heterodimers with AMS, bHLH010, bHLH089, and bHLH091 (Feng et al., 2012).

DYT1 is also a key modulator of the *DEFECTIVE IN TAPETAL DEVELOPMENT AND FUNCTION 1* (TDF1, AT3G28470) transcriptional factor (Figure 3B), and both are expressed at the same stages (stages 4-5) (Figure 1) (Zhu et al., 2011). TDF1 activates the expression of anther development-related genes, including *AMS*, *MYB103* (also known as *MYB80* and *MALE STERILE 188*, *MS188*, AT5G56110), *TEK* (*TRANSPOSABLE ELEMENT SILENCING VIA AT-HOOK*)*,* and *MS1* (Lou et al., 2018). *MYB103* transcripts were detected during stages 6 and 7 (Figure 1) (Zhu *et al*., 2011). The expression of *MS1* occurs in both tapetum and microspores at stages 9 until 12 (Figure 1) (Zhu *et al*., 2011). MS1 acts upstream of multiple pollen coat protein genes and is essential for tapetum development and pollen formation (Lu et al., 2020). However, it remains unclear whether TDF1 binds directly to the promoter of *MS1* to activate its expression and which proteins interact with DYT1 to drive *TDF1* expression itself (Gu et al., 2014).

AMS, induced by TDF1, is another essential bHLH factor for tapetal function and pollen development (Xu et al., 2010). AMS can form homo and heterodimers and bind to the promoter of several genes, therefore directly regulating their function. It plays a central role in pollen wall formation through the direct/indirect activation of genes related to sporopollenin production and pollen coat proteins (Figure 3B) (Xu *et al.*, 2014; Xiong et al., 2016; Lu et al., 2020). AMS can also form heterodimers with TDF1 to activate target gene expressions, such as *MYB103*, enhancing the concept of the feedforward loops that dictate anther development (Lou et al., 2018). AMS exhibits a biphasic protein expression in anther tapetal cells, exhibiting different functions in the early and late stages of pollen development. Ferguson et al. (2017) proposed a model for the network and regulation of AMS, in which the AMS protein and gene expression levels are regulated by MS1. Figure 3C summarizes the feedback loops found in Arabidopsis bHLHs anther development. 

DYT1, AMS, bHLH010/089/091, and other unknown proteins form heterodimers and regulate many genes. DYT1 regulates the expression of *TDF1*, a gene product that regulates the expression of *AMS,* by binding into its promoter (Lou et al., 2018). AMS can regulate the expression of several genes, including *MYB103* and *MS1*. AMS can negatively regulate the expression of *DYT1* and, to a minor extent, *TDF1*. However, it was proposed that MS1 plays a major role in *TDF1* repression (Ferguson et al., 2017). Besides, MS1 indirectly decreases AMS protein levels and represses *AMS* gene expression, potentially by inducing protein degradation and chromatin remodeling, respectively (Figure 3C). The bHLH network regulation is complex, involving intricate mechanisms such as protein-protein interactions, post-translationally modifications, anther stage-specific gene expression patterns, and a multitude of feedforward loops that either activate or repress bHLH transcription factors.

## Conclusions and Perspectives

This review provides a comprehensive overview of the bHLH transcription factors involved in anther development in rice and Arabidopsis. It covers the specific transcription factors involved, their regulatory functions, and the stages at which respective genes are expressed. The review highlights the complexity of the process, as evidenced by the accurate control of expression throughout anther development. Despite significant progress in understanding this regulatory pathway, many aspects remain unexplored. For example, UDT1 is a key bHLH involved in early anther development and regulates TDR and EAT1 (Moon et al., 2020). However, it is still unclear whether UDT1 binds directly to the promoter region of these genes. Recent research has identified new members involved in anther regulatory pathways, such as TIP3 (Yang et al., 2019b). Nonetheless, their regulatory targets, along with TDR, remain undefined. Additionally, the transcription factor OsbHLH035 has recently been implicated in anther development, yet it still requires further investigation (Ortolan et al., 2021). To better understand the complex process of anther development, new studies are needed on the transcriptome analyses of knockout and overexpressing plant lines concerning these genes. Despite significant progress in understanding the role of bHLH transcription factors in anther development, the involvement of other transcription factor families remains understudied. For example, the MYB transcription family appears to play a key role in anther development, but there is still limited understanding of its specific functions in this process. Further studies are necessary to fully elucidate the complex regulatory pathways involved in anther development and identify additional transcription factors involved.

Arabidopsis bHLHs also play essential roles during anther development. Through the interaction among different bHLHs and transcription factors, they create a complex network that dictates which set of genes must be repressed and activated to progress to different stages. These interactions generate feedforward and negative/positive feedback regulatory loops that regulate anther and pollen development (Fu et al., 2020; Lai et al., 2022). It is well established that DYT1 is upstream to TDF1, AMS, MYB103, and MS1 playing a crucial role as the primary transcription factors in tapetum development and function (Lu et al., 2020). Despite the solid knowledge of which regulators are upstream and downstream, there are several open questions about how these proteins regulate themselves. MS1, for instance, can readjust AMS protein levels and repress *AMS* gene expression, but the mechanism behind this remains elusive (Ferguson et al., 2017). Although recent efforts were made to differentiate the function of bHLH010, bHLH089, and bHLH091, it is still not fully clear how they individually contribute to anther development, and whether they contribute to DYT1 translocation to the nucleus at anther stage 5 (Cui et al., 2016; Fu *et al.*, 2020). Additionally, it is unclear whether BES1 and BZR1 can directly regulate *DYT1* expression (Chen et al., 2019). Besides, new studies should focus on searching for other proteins/transcriptional factors that might interact with bHLHs (via heterodimers) and regulate their functions. Approaches such as ChIP-Seq and co-immunoprecipitation, tested in different anther development stages, for instance, would clarify which genes are directly regulated by the aforementioned bHLHs and which proteins make heterodimers with them, respectively (Jamge et al., 2018; Nakato and Sakata, 2021).

Understanding the intricate regulatory mechanisms governing anther development is pivotal for advancing crop yield enhancement and the formulation of novel plant breeding strategies. bHLH transcription factors are also implicated in plant fertility in response to stress. The Cytoplasmic Male Sterility (CMS) system, which employs genetic engineering to induce male sterility in plants through the expression of ribonuclease under a tapetum promoter, facilitates controlled crossing by necessitating the presence of a ribonuclease inhibitor gene in the ‘restauring line’ for fertility restoration (Goldberg et al., 1993; Parish and Li, 2010). UDT1 and TDR are implicated in cold stress, with derepression of these genes in a *wrky53* mutant promoting normal seed setting, suggesting a strategy to enhance productivity under cold conditions (Tang et al., 2022). *AMS* downregulation due to Fe-deficiency affects tapetum formation, potentially alleviated by *AMS* overexpression; additionally, chickpea *AMS* upregulation under salinity stress may confer resistance (Huang and Suen, 2021; Kaashyap et al., 2022). Mutants of *bHLH010*, *089*, and *091* display defective pollen development in response to heat stress, emphasizing their importance in anther development under high-temperature conditions (Fu et al., 2020). In drought and salinity stresses, *OsbHLH035* upregulation is observed, leading to delayed germination rates due to an overaccumulation of abscisic acid (ABA), potentially rendering this gene significant in stress resistance (Chen et al., 2018). These insights offer promising avenues for enhancing plant resistance and productivity.
